# Longitudinal Associations Between Self-reported Schizotypy Dimensions and White Matter Integrity Development During Adolescence

**DOI:** 10.1093/schbul/sbad147

**Published:** 2025-03-04

**Authors:** Mélodie Derome, Suje Amir, Elodie Sprüngli-Toffel, George Salaminios, Eduardo FonsecaPedrero, Martin Debbané

**Affiliations:** Translational Research Center, University Hospital of Psychiatry, University of Bern, Bern, Switzerland; Developmental Clinical Psychology Research Unit, Faculty of Psychology and Educational Sciences, University of Geneva, Geneva, Switzerland; Neuro-X Institute, School of Life Sciences, Swiss Federal Institute of Technology (EPFL), Lausanne, Switzerland; Department of Clinical Neuroscience, Lausanne University Hospital (CHUV) and University of Lausanne (UNIL), Lausanne, Switzerland; Department of Psychiatry, University of Geneva, Switzerland; Department of Psychiatry, Vaud University Hospital Center, Lausanne, Switzerland; Research Department of Clinical, Educational and Health Psychology, University College London, London, UK; Department of Educational Sciences, University of La Rioja, Logroño, Spain; Developmental Clinical Psychology Research Unit, Faculty of Psychology and Educational Sciences, University of Geneva, Geneva, Switzerland; Research Department of Clinical, Educational and Health Psychology, University College London, London, UK

**Keywords:** schizophrenia, neuroimaging, early detection

## Abstract

**Background:**

Alterations of white matter microstructure have been reported in the psychosis spectrum. However, the development of these alterations during preclinical stages remains poorly understood. The framework proposed by schizotypy research as the personality base for liability to develop psychosis spectrum disorders offers 3 interconnected dimensions thought to impact neurodevelopment, affording an opportunity to investigate premorbid risk.

**Design:**

In this study, 102 typically developing individuals aged between 12 and 20 y.o. at baseline were scanned longitudinally between 1 and 4 times, and schizotypy was assessed at each visit. Ten white matter tracts were reconstructed using TRACULA, and mixed model regression was used to characterize age-related changes in main diffusion parameters (ie, fractional anisotropy [FA]). Estimated longitudinal trajectories of the 3 dimensions of schizotypy were tested for different trajectories of diffusion parameters as a function of age.

**Results:**

Positive schizotypy trajectory was the most strongly decreased when FA in the anterior thalamic radiation (atr-FA) increased in young adults compared with a moderate decrease in younger participants. Furthermore, in adolescents, disorganized schizotypy followed a steep increase when atr-FA increased, while in the older participants, it decreased as a function of atr-FA. Independent of age, intraindividual positive schizotypy was further longitudinally negatively associated with FA in the cingulate gyrus, and disorganized schizotypy was positively associated with FA in the superior longitudinal fasciculus.

**Conclusions:**

Given that abnormalities in fronto-thalamo-cingulate subcircuit are present in schizophrenia and converters to psychosis, our results support the hypothesis of schizotypy as a personality base risk to develop psychosis.

## Introduction

Altered cerebral connectivity is one of the core pathophysiological mechanism underlying the development and progression of cognitive deficits in schizophrenia.^1^ Research in the field has led to the hypothesis of a white matter (WM) disrupted connectivity involving insufficient or ineffective communication between brain regions mediating appropriate information processing.^2^ However, accumulating evidence along the spectrum of psychosis, notably at the onset and during the preclinical stages have yielded variable results. The onset of symptoms usually occurs in late adolescence and begins gradually and progresses overtime, between 2 and 5 years before clinical diagnosis.^3,4^ Adolescence is therefore a critical period for the study of psychological traits in psychosis spectrum disorders; it is the stage of social, motor, and cognitive adjustments,^5^ and the onset of puberty involving maturational, hormonal, social, and brain changes.^6^ Seeking to understand the early determinants of risk for psychotic disorders, studying adolescent and early adulthood WM maturation trajectories during the premorbid vulnerability stages of psychosis expression is necessary to ascertain similarities and discrepancies with results from clinical cohorts.

Schizotypy is a useful construct to study schizophrenia spectrum disorders and is described as a latent personality organization reflecting a putative liability to develop psychosis spectrum disorders.^7^ The phenotypic expression of schizotypy, such as schizotypal traits and psychotic-like-experiences may be considered the behavioral expression of increased vulnerability for psychosis.^8^ Schizotypal traits can be measured with the most widely employed questionnaire, the Schizotypal Personality Questionnaire (SPQ),^9^ which yields a 3-factor structure encompassing the positive or cognitive perceptual (paranoid delusions and unusual perceptual experiences), negative or interpersonal (social anxiety, constricted affect, and lack of close friends), and disorganization (odd speech and behaviors) dimensions.^10^

Only 6 studies to date have examined the links between schizotypal traits and WM structural alterations, all restricted to adult samples. (1) Nelson et al. showed that decreased Fractional Anisotropy (FA) values in 4 fronto-temporal WM tracts were associated with increased scores on the SPQ positive dimension in 21 individuals from the general population. Furthermore, a relationship was also found between the same SPQ dimension and FA in 3 controls tracts: left and right anterior thalamic radiations and the forceps minor.^11^ (2) Wang et al. included 87 adults with high schizotypy (HS, SPQtot = 23.43) and found increased structural connectivity between the auditory network and the task control network in the HS group, supporting the hypothesis of vulnerable neural pathways.^12^ (3) DeRosse et al. focused on 67 adult HS scorers (SPQtot = 8.52) and targeted FA in 5 association tracts. Relative to individuals with a low total score (SPQtot = 0.75), HS presented lower FA values in the inferior fronto-occipital fasciculus and greater asymmetry on the right (vs left) side in the uncinate fasciculus,^13^ suggesting that fronto-temporal alterations may represent a core component of psychosis phenotype. (4) Pfarr and Nenadic^14^ used the multidimensional schizotypy scale, and targeted 5 major association tracts of 104 healthy adults. Results showed negative correlations of FA with disorganized schizotypy in the superior longitudinal fasciculus, while negative schizotypy was associated with more widespread effects (positive correlations in uncinate, anterior thalamic radiation, forceps minor, and Inferior fronto-occipital fasciculus). Their results support a fronto-striatal neurobiological continuum model of psychosis. (5) Another study used probabilistic tractography algorithm (PICo) to investigate bilaterally uncinate and arcuate fasciculi. They showed that participants with schizotypal features exhibited increased FA values in the L hemisphere of the uncinate fasciculus only. In the whole sample, there was a positive correlation between FA values and measures of hallucinatory experiences in the right arcuate fasciculus.^15^ (6) Volpe et al. demonstrated that nonclinical participants scoring high on “psychotic personality traits” displayed WM deficits in the corpus callosum, right arcuate (superior longitudinal) fasciculus, and fronto-parietal fibers.^16^

Overall, studies on schizotypy among nonclinical adults have found similarities in terms of WM alterations to previous studies with participants who were on the clinical schizophrenia spectrum, as well as with studies examining individuals with at-risk mental states. Reduced FA values have been observed in groups meeting the Ultra High Risk (UHR) of developing psychosis criterion in the superior longitudinal fasciculus,^17^ yet other studies failed to observe baseline FA differences between UHR who later transitioned to psychosis and those who did not.^18^ Nonetheless, UHR individuals who later transitioned to psychosis showed lower FA than controls in the anterior thalamic radiation and inferior fronto-occipital fasciculus.^19^ Furthermore, in a longitudinal study, UHR patients who went through transition did show subsequent FA reductions in left frontal WM (corona radiata, superior fronto-occipital fasciculus, and anterior body of the corpus callosum) compared with nontransitioned UHR.^20^

Heterogeneity within the literature on WM abnormalities in relation to risk for psychosis may be due to differences in data collection methods (image acquisition), sample characteristics, measurements of schizotypy with the use of various questionnaires,^21^ and analyses methodologies (mainly cross-sectional studies, ROIs vs whole brain, and statistical analyses). Previous investigations of WM alterations and schizotypy have been restricted to adult samples, that are either within or beyond the window of clinical psychosis expression. The present study is, to our knowledge, the first to include the period of adolescence in relation to schizotypy and WM alterations. We examined whether age, WM parameters (FA, RD, AD, and MD) in 10 different tracts, and the interaction between age and WM parameters maturation trajectories significantly predicted the developmental trajectories of schizotypy dimensions. This whole-brain longitudinal study in nonclinical adolescents and young adults may reveal both common WM characteristics with those shown in previous studies among high risk and clinical samples, as well as identify potential compensation characteristics specific to nonclinical samples. We expected significant and specific associations between FA values and the different dimensions of schizotypy in the main association tracts. However, given the large heterogeneity in directionality of FA changes observed in UHR samples, as well as the absence of longitudinal studies examining prospective associations to schizotypy, we could not form specific hypotheses about the effects of WM parameters on the developmental trajectory of schizotypy dimensions.

## Methods

### Participants

One hundred and eleven typically developing French-native adolescents and young adults (59 males, 53 females) were recruited through word of mouth, flyers, and Facebook groups in the context on a longitudinal study carried out at the University of Geneva.^22,23^ Participants were screened from the general population of adolescents and young adults with the aim to study the longitudinal evolution of personality traits during adolescence. They were aged between 12 and 20 years at the first time point of data collection (mean age 17.1, *SD* = 2.4). Each participant or their parents (if they were under 18) provided written informed consent under protocols approved by the local ethical commission (Commission Central d’Ethique de la Recherche des Hopitaux Universitaires de Genève). They received a financial compensation of 100 swiss francs per visit and underwent assessment on their cognitive and psychological functioning as well as magnetic resonance imaging protocol (MRI). Exclusion criteria included mental, psychiatric, or neurological disorders as well as contraindication for MRI assessed with a prescreening questionnaire. Nine participants were excluded: 7 participants suffering from diagnosed anxiety disorders and depression, 1 participant because of a clinical diagnosis, and 1 diagnosed with Attention Deficit Hyperactivity Disorder (ADHD). The final sample included 102 participants (54 males, 48 females). See supplementary material 1a and 1b for more details on participants recruitment.

### Study Design

**Fig. 1.**
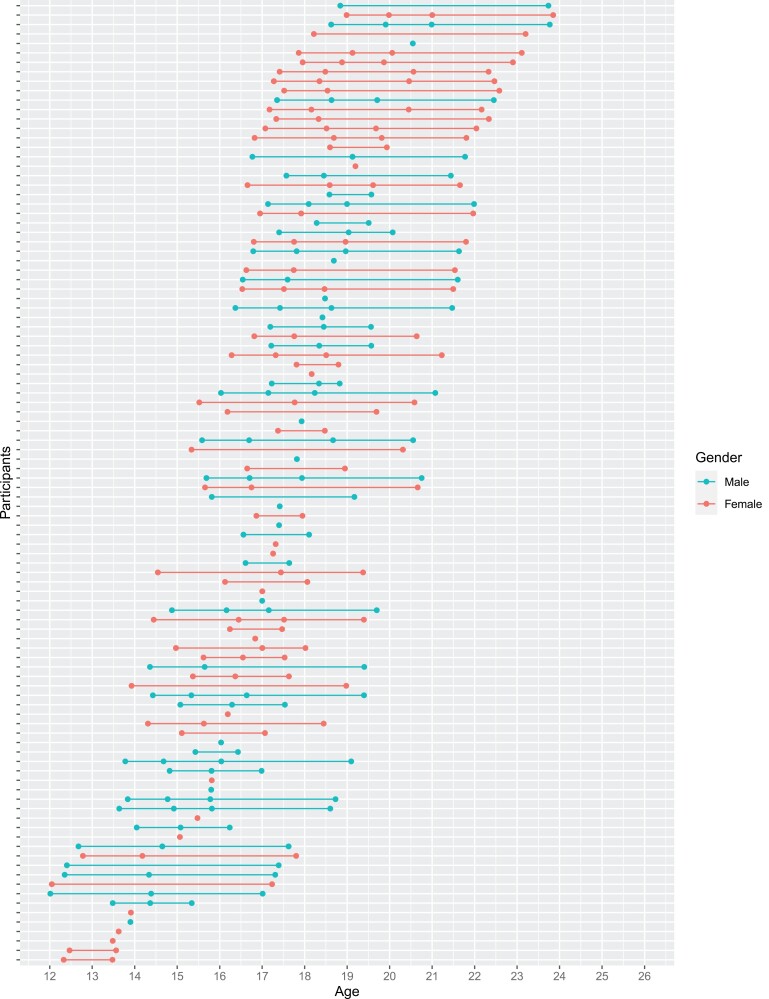
Scan distribution across the age range. *Note.* First point of a continuous line represents the age of participant at baseline (T0). Continuous lines show the longitudinal age of participants, while bullet points represent the timepoint they attended. Single bullet points represent participants who came for only 1 visit.

The present study followed a longitudinal within-subject design. Schizotypy, MRI scans, and covariate measures were acquired at 1–4 visits per each participant over the course of 4 years, with the following intervals: 1 year between baseline and second visit (T0 and T1), 1 year between the second and third visit (T1 and T2), and 2 years between the third and fourth visit (T2 and T4) due to the funding schedule. The number of assessments varied between participants, a total of 259 scans were acquired, comprising 25 adolescents with 1, 23 with 2, 28 with 3, and 26 with 4 scans, see figure 1 Overall, the longitudinal sample consisted of 89 participants at T0 (mean age = 15.9), 73 at T1 (mean age = 17.5), 54 at T2 (mean age = 18.4), 43 at T4 (mean age = 20.8). See table 1.

**Table 1.** Descriptive Statistics of Demographic and Questionnaire Measures at All Measurement Time Points

	Baseline (T0)		T1		T2		T4		statistics
*N*	89		73		54		43		-
Age	15.9(1.80)		17.5(1.78)		18.4(1.82)		20.8(1.81)		*F*(3) = 82.839, *P* < .001
SPQ positive	9(7.19)		6.82(5.99)		5.26(5.19)		5.37(5.24)		*F*(3) = 5.324, *P* = .001
SPQ negative	6.34(4.48)		5.56(4.33)		5.39(3.68)		5.79(4.21)		*F*(3) = 0.942, *P* = .421
SPQ disorganized	5.83(3.83)		5.51(3.82)		4.41(3.51)		5.09(3.50)		*F*(3) = 1.731, *P* = .161
Sex	Females *n* = 43	Males *n* = 46	Females *n* = 33	Males n = 40	Females *n* = 28	Males *n* = 26	Females *n* = 21	Males *n* = 22	*χ*²=0.878
Vocabulary	11.3(3.08)		10.7(3.42)		10.5(3.34)		10.6(2.93)		*F*(3) = 1.083, *P* = .357
Block design	10.7(3.09)		11.5(3.59)		11.6(3.48)		12.2(3.24)		*F*(3) = 2.305, *P* = .077
Average (vocabulary + Block design)	11(2.57)		11.1(2.95)		11.1(2.90)		11.4(2.61)		*F*(3) = 0.361, *P* = .781
Internalizing total score	53.1(10.5)		50.8(8.5)		51.7(10.9)		53.9(10.1)		*F*(3) = 1.172, *P* = .321
Externalizing total score	55.8(9.31)		56(9.25)		54.3(8.24)		53.9(8.91)		*F*(3) = 0.870, *P* = .457

*Note.* Values indicate mean(S*D*) scores assessed at a given timepoint, unless specified otherwise. Vocabulary and Block design represent the subtests of Wechsler’s WAIS/WISC-IV.

All values were computed from unstandardized scores for descriptive purposes.

### Measures

#### Questionnaires and Neuropsychological Tests.

All participants completed questionnaires to assess cognitive functioning, adaptive behaviors, and schizotypal personality traits (SPQ),^24^ see supplementary material 1a, 1c, 1d, 1e for details.

#### MRI Measures.

Preprocessing of the T1 images was conducted using Freesurfer software version 6.0 (https://surfer.nmr.mgh.harvard.edu), see supplementary material 1f. This procedure has been described previously in.^22, 23, 25^

Subsequently, we used the longitudinal pipeline of the TRacts Constrained by Underlying Anatomy (TRACULA) tool from Freesurfer v6.0 to reconstruct 18 (right and left) major WM paths in each subject’s timepoints,^26^ see supplementary material 1g. Available tracts included: corpus callosum forceps major (fmaj), and forceps minor (fmin), anterior thalamic radiation (atr), Corticospinal tract (cst), superior longitudinal fasciculus parietal (slfp) and temporal (slft), inferior longitudinal fasciculus (ilf), cingulum-cingulate gyrus bundle (ccg), cingulum angular bundle (cab), and uncinate fasciculus (unc). The automated longitudinal global probabilistic tractography algorithm estimates the probability distribution of WM tracts given the T1 and DTI information from all timepoints. TRACULA performs tract reconstruction in the native space of the subject to ensure that same WM paths are compared between time points to increase the sensitivity to longitudinal changes. Once tracts distribution have been estimated, TRACULA extracts 4 diffusion measures as averaged per tract: (1) FA indicates the fraction of diffusion that is directionally constrained; (2) radial diffusivity (RD) represents the amount of diffusion perpendicular to the main direction; (3) axial diffusivity (AD) is the main direction of diffusion; and (4) mean diffusivity (MD) reflects the magnitude of diffusivity in the tissue. FA and MD were analyzed as primary outcomes of interest. RD and AD were analyzed in any tracts where FA and or MD showed significant results, in order to investigate which WM factors may influence the results.

### Statistical Analyses

All analyses were conducted using JAMOVI software (https://www.jamovi.org). Age-related changes of FA, MD, RD, and AD in relation to schizotypy were analyzed using mixed models to account for the repeated measurements of each individual. Mixed model analysis can handle unequal numbers of within-subject measurements and it accounts for interindividual variability. Starting from a baseline null model, models of increasing complexity were iteratively added in a stepwise fashion, see supplementary table 1 for model comparison. Model comparisons were conducted between the current and next model in the iteration using a combination of fit statistics. First, we compared the Akaike Information Criterion between models, with lower values indicating a better fit between the observed and true model.^27^ All continuous predictors were mean centered to improve interpretability of the intercepts. Our final model included age and diffusion parameters per tracts as the fixed-effects factors, subjects as a random-effect factor, and schizotypy as dependent variable. We conducted 3 separate models (one for each dimension of schizotypy), firstly with FA, and further replacing FA with MD, and then AD and RD only in significant tracts.^28^ Restricted maximum likelihood estimation was set to true based on prior recommendations.^29^ Genders were combined for the analyses, but a sex term was included to account for any absolute differences based on gender. The age2 and age3 terms represented quadratic and cubic fit. If neither were significant, a linear fit was calculated. Averaged values for both right and left hemisphere were combined, and 1 fit was done for both sides. Level of statistical significance was set at *P* < .05 (two-tailed). Models’ equation can be found in the supplementary material 1h.

## Results

### Age-related Changes and Gender Differences in Diffusion Parameters

Using mixed models, we first tested the effect of age (linear, quadratic, and cubic), gender, and their interaction on each WM tracts diffusion parameters. First, we tested each tracts’ FA as dependent variable, we further computed the same models for MD, AD, and RD in tracts showing significant effects. FA of the anterior thalamic radiation (atr_FA) showed a linear decrease as a function of age (*t*[222] = –5.456, *P* < .001), while there was no effect of gender (*t*[232] = –0.586, *P* = .558), or of the interaction age*gender (*t*[222] = 0.809, *P* = .419). atr_MD, atr_AD and atr_RD showed increasing linear trajectories with age (respectively; MD: *t*[238] = 0.5.850, *P* < .001; AD: *t*[222] = 5.101, *P* < .001; RD: *t*[233] = 5.730, *P* < .001). No gender or interaction effect was found for MD, AD, and RD either. See supplementary figure 1 and supplementary table 2.

### Age-related Changes in Schizotypy

The negative dimension of schizotypy did not yield significant association with age, see Supplementary table 3.

Positive and disorganized schizotypy exhibited significant linear decreasing trajectories with age (respectively: *t*[216] = −5.55, *P* < .001 and *t*[222] = –2.0230, *P* = .044). In addition, females showed on average higher levels of positive (*t*[227] = –2.17, *P* = .03) and disorganized (*t*[231] = –2.390, *P* = .018) schizotypy than males. The interaction between gender and age had a significant effect on disorganized schizotypy; while females exhibited a linearly decreasing trajectory with age, male showed a slight increase trajectory of disorganized schizotypy in our sample (*t*[220] = 2.055, *P* = .041). See supplementary table 3 and supplementary figure 2.

### Diffusion Parameters Maturation and Schizotypy

No significant interactions between negative schizotypy and diffusion parameters maturation trajectories were found.

**Fig. 2.**
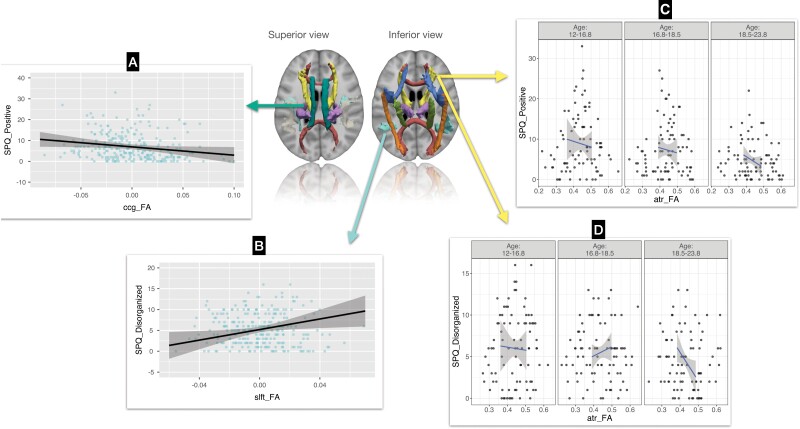
Schizotypy trajectories by diffusion parameters and age. *Note*. Images of the tracts represent the TRACULA reconstruction for 1 participant, overlayed on their T1 anatomical image. A and B represent the interaction between FA diffusion parameter and schizotypy: (**A)** positive schizotypy, and (**B)** Disorganized schizotypy. The y-axis reflects schizotypy scores changes over time. C and D represent the effect of interaction between age and FA on the developmental trajectory of schizotypy: (**C)** for positive schizotypy, and (**D)** for disorganized schizotypy. ccg stands for cingulum-cingulate gyrus bundle, slft for superior longitudinal fasciculus temporal, and atr for anterior thalamic radiation.

Positive schizotypy showed a significant negative association with FA in the cingulum-cingulate gyrus bundle (ccg_FA, *t*[201.6] = –2.920, *P* = .004) while ccg_MD (*t*[207.5] = 2.026, *P* = .044) and ccg_RD (*t*[208.1] = 2.750, *P* = .006) increased when positive schizotypy increased. See table 2 and figure 2A.

**Table 2.** Interaction Between Age and Diffusion Parameters on the Developmental Trajectory of Positive Schizotypy

			95% Confidence interval				
Names	Estimate	SE	Lower	Upper	*df*	*t*	*P*
(Intercept)	6.66	0.600	5.4885	7.839	75.6	11.114	<.001
**Age**	**−1.435**	**0.3343**	**−2.0899**	**−0.779**	**69.6**	**−4.2908**	**<.001**
Age2	0.8295	0.5318	**−**0.2129	1.8718	122.1	1.55964	.121
Age3	**−**0.0160	0.8059	**−**1.5956	1.5636	155.6	**−**0.01984	.984
fmajorFA_Avg	8.455	11.6911	**−**14.4592	31.369	192.0	0.7232	.470
fminorFA_Avg	**−**17.404	12.7515	**−**42.3961	7.589	195.7	**−**1.3648	.174
atr_FA	22.410	22.1024	**−**20.9095	65.730	195.9	1.0139	.312
cab_FA	**−**17.000	10.3992	**−**37.3821	3.382	205.4	**−**1.6347	.104
**ccg_FA**	**−47.066**	**16.6227**	**−79.6454**	**−14.486**	**184.5**	**−2.8314**	**.005**
**ccg_MD**	**51927**	**25624**	**1703**	**102150**	**207.5**	**2.026**	**.044**
**ccg_RD**	**40936**	**18287.7**	**5092.7**	**76779**	**215.3**	**2.238**	**.026**
cst_FA	2.002	19.6519	**−**36.5153	40.519	199.6	0.1019	.919
ilf_FA	14.921	20.9715	**−**26.1826	56.024	191.5	0.7115	.478
slfp_FA	**−**13.761	32.3345	**−**77.1358	49.613	189.5	**−**0.4256	.671
slft_FA	40.217	38.5636	**−**35.3659	115.801	191.2	1.0429	.298
unc_FA	10.064	20.4175	**−**29.9532	50.082	202.0	0.4929	.623
Gender	**−**0.909	1.0712	**−**3.0086	1.190	82.7	**−**0.8487	.399
Average (Block-Voc)	**−**0.316	0.167	**−**0.644	0.0123	230	**−**1.886	.060
**Internalizing**	**0.136**	**0.0378**	**0.0616**	**0.210**	**193.7**	**3.5905**	**<.001**
**Externalizing**	**0.162**	**0.0448**	**0.0743**	**0.250**	**208.5**	**3.6180**	**<.001**
Age ✻ fmajorFA_Avg	**−**18.343	13.0561	**−**43.9321	7.247	96.5	**−**1.4049	.163
Age ✻ fminorFA_Avg	23.941	14.6355	**−**4.7445	52.626	108.3	1.6358	.105
**Age** ✻ **atr_FA**	**−41.133**	**21.3029**	**−82.8856**	**0.620**	**87.0**	**−1.9308**	**.042**
**Age ✻ atr_RD**	**27176**	**13771**	**184**	**54168**	**84.8**	**1.973**	**.047**
Age ✻ cab_FA	9.502	9.9501	**−**10.0002	29.003	77.7	0.9549	.343
Age ✻ ccg_FA	7.284	12.8804	**−**17.9612	32.529	67.6	0.5655	.574
Age ✻ cst_FA	**−**23.738	17.9574	**−**58.9340	11.458	76.8	**−**1.3219	.190
Age ✻ ilf_FA	11.614	17.5598	**−**22.8026	46.031	66.1	0.6614	.511
Age ✻ slfp_FA	**−**12.654	28.3178	**−**68.1553	42.848	71.8	**−**0.4468	.656
Age ✻ slft_FA	36.826	32.5634	**−**26.9969	100.649	59.3	1.1309	.263
Age ✻ unc_FA	**−**0.272	16.9094	**−**33.4142	32.869	70.4	**−**0.0161	.987

*Note*. The model includes Age, Gender, Average (Block design—Vocabulary) subtests, internalizing, and externalizing as covariates. fmaj represents the corpus callosum forceps major, fmin, the forceps minor, cst the cortico-pinal tract, slfp the superior longitudinal fasciculus parietal, slft the temporal, ilf the inferior longitudinal fasciculus, ccg the cingulum-cingulate gyrus bundle, cab the cingulum angular bundle, unc the uncinate fasciculus. SE stands for standard error.

On the other hand, disorganized schizotypy was associated positively with FA in the superior longitudinal fasciculus temporal (slft_FA, *t*[226.2] = 2.0505, *P* = .041), while slft_RD showed a negative association (*t*[227.5] = –2.3714, *P* = .019) with disorganized schizotypy. See table 3 and figure 2B.

**Table 3.** Interaction Between Age and Diffusion Parameters on the Developmental Trajectory of Disorganized Schizotypy

			95% Confidence interval				
Names	Estimate	SE	Lower	Upper	*df*	*t*	*P*
(Intercept)	5.2567	0.335	4.600	5.9136	95.1	15.6840	<.001
**Age**	**−0.5025**	**0.219**	**−0.931**	**−0.0738**	**77.2**	**−2.297**	**.024**
Age2	0.1786	0.366	**−**0.538	0.895	128.8	0.4882	.626
Age3	**−**0.0214	0.553	**−**1.106	1.063	157.2	**−**0.0387	.969
fmajorFA_Avg	7.9269	7.4984	**−**6.7698	22.624	197.0	1.05714	.292
fminorFA_Avg	**−**9.7380	8.1384	**−**25.6888	6.213	199.7	**−**1.19655	.233
atr_FA	3.5294	13.9241	**−**23.7613	30.820	190.9	0.25347	.800
cab_FA	0.7006	6.6332	**−**12.3003	13.701	205.4	0.10561	.916
ccg_FA	**−**12.1300	10.4228	**−**32.5583	8.298	177.3	**−**1.16379	.246
cst_FA	**−**22.5579	12.4203	**−**46.9012	1.785	196.3	**−**1.81622	.071
ilf_FA	3.1931	13.2028	**−**22.6839	29.070	183.5	0.24185	.809
slfp_FA	**−**38.8498	20.3867	**−**78.8069	1.107	186.7	**−**1.90565	.058
**slft_FA**	**55.7963**	**26.627**	**3.609**	**107.9840**	**198.4**	**2.095**	**.037**
**slft_RD**	**−58918**	**28440**	**−114661**	**−3176**	**213.2**	**−2.0717**	**.040**
unc_FA	15.7016	12.9131	**−**9.6077	41.011	196.0	1.21594	.225
Avg (VocBlock)	0.0859	0.0922	**−**0.0948	0.267	186.7	0.93184	.353
Gender	**−**0.1178	0.6624	**−**1.4160	1.181	86.6	**−**0.17779	.859
**Internalizing**	**0.0823**	**0.0240**	**0.0353**	**0.129**	**197.0**	**3.42990**	**<.001**
**Externalizing**	**0.1052**	**0.0284**	**0.0495**	**0.161**	**205.9**	**3.70191**	**<.001**
Age ✻ fmajorFA_Avg	**−**8.4395	8.4342	**−**24.9702	8.091	108.0	**−**1.00063	.319
Age ✻ fminorFA_Avg	4.8722	9.4865	**−**13.7210	23.466	118.8	0.51360	.608
**Age** ✻** atr_FA**	**−34.2265**	**14.174**	**−62.006**	**−6.4468**	**99.2**	**−2.415**	**.018**
**Age** ✻** atr_RD**	**18712**	**8703**	**1654**	**35769**	**89.7**	**2.1500**	**.034**
Age ✻ cab_FA	**−**1.6961	6.4413	**−**14.3207	10.929	87.2	**−**0.26331	.793
Age ✻ ccg_FA	6.0422	8.3586	**−**10.3404	22.425	77.6	0.72287	.472
Age ✻ cst_FA	8.6859	11.6418	**−**14.1316	31.503	86.5	0.74609	.458
Age ✻ ilf_FA	6.3411	11.4090	**−**16.0201	28.702	75.9	0.55580	.580
Age ✻ slfp_FA	**−**5.7746	18.3421	**−**41.7245	30.175	82.1	**−**0.31483	.754
Age ✻ slft_FA	6.7100	21.0809	**−**34.6079	48.028	69.0	0.31830	.751
Age ✻ unc_FA	**−**0.0225	10.9761	**−**21.5353	21.490	80.4	**−**0.00205	.998

*Note.* The model includes Age, Gender, Average (Block design—Vocabulary) subtests, internalizing, and externalizing as covariates. fmaj represents the corpus callosum forceps major, fmin, the forceps minor, cst the cortico-pinal tract, slfp the superior longitudinal fasciculus parietal, slft the temporal, ilf the inferior longitudinal fasciculus, ccg the cingulum-cingulate gyrus bundle, cab the cingulum angular bundle, unc the uncinate fasciculus. SE stands for standard error.

### Interaction Between Age and Diffusion Parameters on the Developmental Trajectory of Schizotypy

Interaction between age and diffusion parameters on the developmental trajectory of negative schizotypy did not yield any significant results, see supplementary table 4.

Positive schizotypy followed an overall decreasing trajectory when FA in the anterior thalamic radiation increased (atr_FA, *t*[84.2] = –2.624, *P* = .010), notably with a steeper decrease at an older age (Mean + 1 *SD*; 18.5–23.8 y.o.) compared with a more steady decrease in younger individuals (12–18.5 y.o.). See table 2 and figure 2C. Positive schizotypy followed steeper increase when atr_RD increased in older individuals, while at younger ages, the increase was more gentle (*t*[112.4] = 3.2374, *P* = .002).

Furthermore, in younger individuals (12–16.8 y.o.), disorganized schizotypy followed a steady decrease when FA increased in the ATR, while in young adolescents (16.8–18.5 y.o.), the trajectory increased abruptly, to then follow a steeper decrease in older individuals (18.5–23.8 y.o.) (atr_FA, *t*[67.6] = −2.5319, *P* = .014). See table 3 and figure 2D. Similarly, disorganized schizotypy keep decreasing steadily as a function of atr_RD in youth, followed by a decrease in adolescents, and an abrupt increase in older ages (*t*[89.7] = 2.1500, *P* = .034). Inclusion of covariates in all analyses is detailed in supplementary material 4b.

## Discussion

The present study investigated whether WM parameters (FA, RD, AD) in 10 different tracts, age, and the interaction between age and WM parameters maturation trajectories significantly predicted the developmental trajectories of schizotypy. Firstly, we confirmed previous hypothesis of linear decreasing trajectory of both positive and disorganized schizotypy with age, we discussed this results in supplementary material 6. Secondly, independent of age, positive schizotypy (eg, unusual perceptual experiences) was negatively associated with FA in the cingulum-cingulate gyrus bundle (ccg), and disorganized schizotypy was positively associated with FA in the superior longitudinal fasciculus temporal (slft). Lastly, positive, and disorganized schizotypy showed different trajectories in the anterior thalamic radiation (atr) as a function of age. Positive schizotypy was most strongly decreased when atr_FA increased in young adults compared with a moderate decrease in younger participants. In adolescents, disorganized schizotypy followed a steep increase when atr_FA increased, while in the older participants, levels of disorganized schizotypy decreased as a function of atr_FA. The resulting WM trajectories in association with schizotypy will be compared with previous findings examining both normative developmental maturation in the general population, as well as with early stages of the schizophrenia spectrum, notably to clinical high-risk samples who converted to psychosis.

### Diffusion Parameters Maturation and Schizotypy

In the present study, positive schizotypy showed a significant negative association with FA in the cingulum-cingulate gyrus bundle. Thus, in other words, higher scores of positive schizotypy are associated with decrease in ccg-FA. In the general population, normative FA maturation in the cingulum follows an increasing trajectory from 5 to 15 y.o and then stabilizes until 30 y.o.^30^ or start decreasing after peaking at 20 y.o. according to Lebel and Beaulieu.^31^ Therefore, our results are going in the opposite direction than normative WM maturation. However, Nelson et al.,^11^ also identified a negative relationship between cognitive-perceptual schizotypy features and FA in the Left cingulum. Along the psychosis continuum, lower FA was also reported in the anterior cingulum of schizotypal personality disorder compared with controls.^32^ Furthermore, FA reduction in the cingulate bundle was observed in CHR individuals (vs controls).^33,34^ Thus, alterations of the ccg appear to contribute to the vulnerability of developing psychosis and might be attributable to abnormal development, especially given than the ccg continues to develop past the typical age of schizophrenia onset.^35^ Interestingly, the cingulum bundle is a major fiber tract of the limbic system that joins the frontal lobe to the parahipocampal gyrus in the medial temporal lobe, suggesting a key role emotional regulation and social cognition.^36,37^ These results complement our previous studies on gray matter trajectories of the same participants showing alterations in the cingulate cortex and hippocampus subcortical structure.^22,23^

On the other hand, data showed that disorganized schizotypy was associated positively with FA in the superior longitudinal fasciculus temporal, suggesting that higher disorganized schizotypy was associated with increased FA in the slft. In the general population, normative WM maturation in the slf shows a steep increase between 5 and 10 y.o. and plateaus after 15 y.o. in Chen et al.^30^ or starts decreasing slightly after 25 y.o. in Lebel and Beaulieu.^31^ Thus, it seems that slft_FA in individuals with higher disorganized schizotypy does not stabilize as observed in the general population, but instead keeps increasing. It must be noted that Pfarr and Nenadic reported a negative association between disorganized schizotypy and FA in the slf,^14^ which goes against the direction of our results, however, their sample only included adult participants. Finally, a previous study reported that UHR individuals who converted to clinical psychosis also showed reductions in FA in the slf.^20^ In this case we could hypothesize that the increase in FA in the slf associated with disorganized features observed during adolescence might constitute a compensatory/protective mechanism. The slf is the largest associative fiber bundle system and lays connections between the frontal and parietal lobes.^38^ Reinforcing our hypothesis, research has showed that FA in the slf predicted improvement of response to therapeutic interactions such as social skills training and cognitive remediation in schizophrenia patients.^39^ The positive association between disorganized schizotypy and FA_slft was also sustained by a significant negative association with RD_slft. RD represents the averaged amount of apparent diffusion along secondary and tertiary diffusion axes, while FA is a relative measure of diffusion anisotropy.^28^ Based on conventional interpretation, we could infer that higher FA value might reflect increased number of microstructural tissue element, however, due to the presence of crossing fibers, disproportionate atrophy (while preservation of other fiber bundles) could result in increase of FA, despite an actual decreased myelination.

### Interaction between Age and FA in the Anterior Thalamic Radiation on the Developmental Trajectory of Schizotypy

Both positive and disorganized schizotypy features were influenced by the interaction effect of age and FA in the anterior thalamic radiation (atr). Positive schizotypy followed an overall decreasing trajectory when atr_FA increased, notably with a steeper decrease at an older age (18.5–23.8 y.o.) compared with a steadier decrease in younger individuals (12–18.5 y.o.). Furthermore, in younger individuals (12–16.8 y.o.), disorganized schizotypy followed a steady decrease when atr_FA increased, while in young adolescents (16.8–18.5 y.o.), the trajectory increased abruptly, to then follow a steeper decrease in older individuals (18.5–23.8 y.o.).

Nelson et al.^11^ identified a negative association between FA in the right and left atr and cognitive-perceptual factor of schizotypy. The present findings confirmed their result, while adding the dimension of age, showing that the decreasing trajectory is steeper in older individuals. No other studies have shown the link between disorganized schizotypy and atr_FA. The longitudinal aspect of the study and the inclusion of the period of adolescence might explain the discrepancies with existing literature. However, previous studies suggest that UHR individuals who later transitioned to psychosis showed lower FA than controls in the atr.^19^ Therefore, we could hypothesize that the developmental decrease of FA in the atr associated with heightened positive schizotypal features might represent a potential early predictive risk marker for the development of psychosis. Conversely the abrupt increase in FA during adolescence in the atr associated with increased disorganized schizotypy might be the result of a compensatory mechanism preventing the development of schizophrenia spectrum disorders. The atr is another major fiber tract linking the thalamus to the prefrontal cortex, with a role in working memory and executive functions.^40^

### Neurobiological Basis of WM Parameters

Previous research suggested that reductions in RD are indicative of excessive myelination^41^ of axonal tracts, whereas reductions in AD may be driven by reduced tract organization, axonal reduction, or axonal diameter reductions.^41–43^ While increases in FA have been suggested to reflect axonal branching (leading in turn to a reduced amount of fiber crossing). Furthermore, FA and RD show an interdependence, as WM bundles become more anisotropic when myelination is increased (RD is reduced^43^). Accordingly, our results suggest that subtle abnormalities linked to positive schizotypy may be characterized by a combination of reduced myelination and increasing amount of fiber crossing in the cingulum and anterior thalamic radiation (reduced FA and increased RD). On the contrary, those associated with disorganized features seem to involve processes of excessive myelination and axonal branching in the superior longitudinal fasciculus (increased FA and reduced RD), however in the atr, we observed the same pattern only during adolescence.

The present data provide evidence that, similarly to the dimensions of psychosis, the positive and disorganized features of schizotypy personality within a nonclinical population have an identifiable neurobiological basis, notably including association tracts. We observed abnormalities in the developmental trajectories of a fronto-limbic subcircuit linked to subtle developmental changes reflecting a neural signature at the nonclinical level.

## Limitations

Some limitations should be taken into consideration. First, while this is the first study to delineate WM developmental trajectories using longitudinal data with up to 4 time points in schizotypy, larger longitudinal samples including a higher number of time points and a longer total follow-up time will be needed to confirm the results. Notably, inclusion of a longitudinal sample that will include converters to psychosis could help discover developmental processes that may be used as risk markers for the development of psychosis-spectrum disorders. Secondly, we used the SPQ, which is a self-report instrument to measure schizotypy. Future studies could benefit by also including an observer-rated assessment tool of schizotypy. Additionally, we only assessed whether adolescents’ substance use (alcohol, cannabis) had consequences on their responsibilities with school or the law. Although we did not exclude any of the participants for such problems, we did not assess cannabis and alcohol quantity, these behaviors are prevalent in adolescents and known to affect MRI-based metrics as well as neurodevelopment and risk for psychosis. Furthermore, we did not screen participants for family history, thus we cannot exclude that some of them had past family history of psychiatric disorders. Finally, the DTI model of diffusion might be suboptimal in assessing complex brain regions with fiber crossing, although widely used and accepted as a valid model for assessment of WM microstructure in human brains.

## Conclusion

Findings of this study are suggestive of an association between psychosis risk phenotype as measured with schizotypy and the fronto-thalamo-cingulate circuit during adolescence. Abnormalities in fronto-thalamo-cingulate subcircuit are present in schizophrenia and converters to psychosis; our findings suggest that such alterations may stem from the schizotypy personality base signifying risk to develop schizophrenia. Specifically, we identified positive personality traits of schizotypy associated with developmental trajectories of FA in the cingulum bundle and anterior thalamic radiation and found similar pattern to those identified along the spectrum of psychosis. We also observed differences in trajectory pattern to those seen along the spectrum, notably in the superior longitudinal fasciculus and anterior thalamic radiation in relation to disorganized schizotypy, which should be further investigated in clinical population to be interpreted as potential protective compensatory mechanism.

## Supplementary Material

Supplementary material is available at https://academic.oup.com/schizophreniabulletin/.

sbad147_suppl_Supplementary_Materials_1-6_Figures_1_Tables_2-4

## Data Availability

Data can be shared upon request.
