# Supplementary material for: Longitudinal Associations Between Self-reported Schizotypy Dimensions and White Matter Integrity Development During Adolescence
Source: Schizophr Bull. 2025 Mar 4;51(Suppl 2):S126–36. doi: 10.1093/schbul/sbad147 (PMC11879505; doi:10.1093/schbul/sbad147)
Supplement: sbad147_suppl_Supplementary_Materials_1-6_Figures_1_Tables_2-4 [file sbad147_suppl_supplementary_materials_1-6_figures_1_tables_2-4.pdf]

Supplementary material

1 Methods - additional information

1.a. Exclusion criteria

Exclusion criteria included: acute psychotic phase, and estimated IQ scoring below 1 std.dev of the developmental norm (based on the Cubes and Vocabulary subtest of the Weschler Scales of Intelligence for children (WISC-IV)[1] or for participants older than 18 y.o, the Wechsler Adult Intelligence Scale[2] (WAIS-IV)). Screening was made through medical questionnaire asking for any previous psychiatric diagnosis, treatment, epilepsy, neurological disorders. We also ask them whether they are following or have followed in the past any medication. In addition, they are asked whether they consult or have consulted psychiatrists or psychologists, or speech therapists.

On this basis we excluded from the present study: 7 participants who suffered from diagnosed anxiety disorders and depression, 5 out of 7 were following psychopharmacological medication, notably for depressive symptoms (Cytralex and Sertraline). One other participant was diagnosed for ADHD and was on medication (Ritaline). One presented schizoaffective disorder combined with a neurological disorder and was thus also excluded from the sample. None of our participants had experienced epilepsy. None of our participant followed psychoactive treatment. Questions concerning substance and alcohol use were asked as well in the medical screening questionnaire. However, we only asked whether their consumption led to incompatibility with responsibilities (i.e. school) or to problems with the law, none were excluded because of alcohol or substance abuse.

1.b. Socio demographic characteristics of the study participants

**Ethnicity**									
Categories	1 = Swiss	2 = French	3 = Southern Europe	4 = North Africa	5 = South Africa	6 = Asia	7 = Mixed	8 = Others	Missing data
N	35	9	7	0	3	0	29	13	11
**Parents's Socio-economic status**									

Proffesional category	1= Dirigeants, senior executive ..	2 = Academic and scientific professions	3 = Intermediate professions	4 = Administrative employés	5 = Customer service and sales personnel	6 = Farmers, hunters …	7 = Machine and industrial robots operators, crane operators, drivers … engib	8 = Workers and unskilled workers	Missing data
N	18	17	25	17	14	5	9	0	2
Formation level	1 = University & higher education	2 =Superior Proffesional schools	3 = A level	4 = Vocational school	5 = Apprenticeship	6 = compulsory school	7 =Less than compulsory school	8 = other	Missing data
N	53	4	9	4	19	15	2	0	1

1.c. Schizotypal Personality Questionnaire

The SPQ measures a broad range of schizotypal traits based on the main features of DSM-3 SPD criteria (DSM-5, 2013). The 74-items are distributed across 9 subscales, each containing 7-9 items (odd beliefs, unusual perceptual experiences, ideas of reference, paranoid ideation, excessive social anxiety, no close friends, constricted affect, odd or eccentric behavior, and odd speech) grouped in 3 dimensions: positive, negative, and disorganized.

1.d. Cognitive functioning

Cognitive functioning was measured at each timepoint using the average standardized scores of the Block Design and Vocabulary subtests of the Wechsler Scales of Intelligence for Children (WISC) for children or the Wechsler Adult Intelligence Scale (WAIS) for adults over 18 years (WAIS-IV; Wechsler, 1997; WISC-IV; Wechsler, 2003). The average scores between block design and vocabulary were used to control for cognitive functioning as covariates of no interest in the following statistical analysis.

1.e. Adaptive behaviors

Adaptive functioning and problems were assessed at each measurement timepoint using the Youth Self report questionnaire (YSR, Bordin et al., 2013) and Adult Self report questionnaire [3]. Thereby, obtained internalizing (withdrawal, anxiety, somatic complaints, and depression) and externalizing (aggressive behaviours, delinquency, and attention problems) scores were used as covariates of no interest to further isolate the specific contribution of schizotypy.

1.f. Preprocessing of T1 images

Structural MRI scans were acquired on a 3-Tesla Siemens Trio scanner, with the following parameters: TR=2500ms, TE=3ms, flip angle=8degree, acquisition matrix=256x256, slice thickness=1.1mm, field of view=22cm, number of slices acquired=192.

Following the fully automated pipeline for three-dimensional cortical model images [4,4]. White matter and pial surfaces were visually checked and manually corrected if necessary for each participant. A longitudinal processing step was added to reduce within-subject variability by creating a within-subject unbiased template and an average image using inverse consistent registration [5].

1.g. Preprocessing of DTI images

DTI images were acquired with a number of direction of 30, b=1000s/mm^2, TE=84ms, TR=8800ms, flip angle=90 degrees, field of view=25.6cm, slice thickness=2mm, acquisition matrix= 128x128, 64 axial slices, GRAPPA acceleration=2.

Main steps included eddy current distortion correction, reorientation of gradient vectors, intra-subject registration, inter-subject registration, brain mask extraction, head motion computation (no subjects' scans were excluded for excessive head motion: > 2 degrees rotation or 2mm translation), and fitting of DTI model.

1.h. Model Equations

The final model equations fitted to the data were as follow:

Model 1: Linear mixed model

SPQ_dimension ~ 1 + Age + FA_Avg + sex + Age:FA_Avg +(1 | Subject_ID)+(0+Age | Subject_ID)

Model 2: Mixed model with age polynomial

SPQ_dimension ~1 + Age + FA_Avg + sex + Age:FA_Avg + I(Age^2) + I(Age^3) + I(Age^2):FA_Avg + I(Age^3):FA_Avg + (1 | Subject_ID) + (0+Age | Subject_ID)

2. Model Comparisons

Supplementary Table 1. Step wise implementation of mixed model analyses and model comparison

	**Model 1 (null)**	**Model 2 (fixed slope)**	**Model 3 (random slope)**
**Positive schizotypy**			
AIC	1615	1594	1418
BIC	1625	1575	1360
R-squared	0.597	0.691	0.731
**Negative schizotypy**			
AIC	1402	1413	1408
BIC	1413	1398	1396
R-squared	0.611	0.623	0.715
**Disorganized Schizotypy**			
AIC	1343	1343	1343
BIC	1354	1331	1334
R-squared	0.587	0.643	0.676

note. Starting from a baseline null model (Model 1), models of increasing complexity were iteratively added in a stepwise fashion (Model 2 then Model 3). AIC stands for Akaike Information Criterion. BIC stands for Bayesian Information Criterion. The smaller the value of AIK and BIC, the better fit is the model.

3. Age-related changes and gender differences in diffusion parameters

Supplementary Figure 1

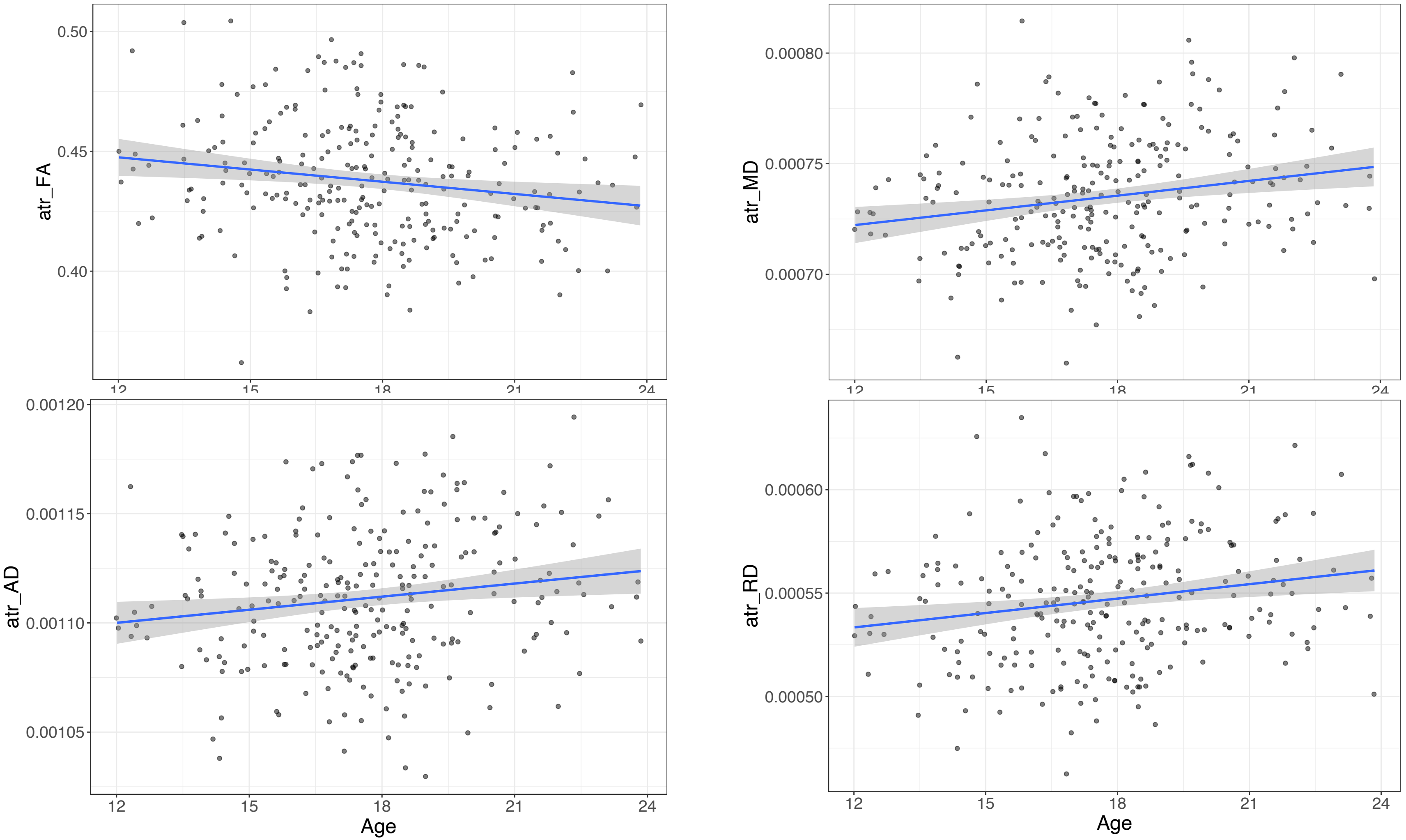

atr_FA

Names	Effect	Estimate	SE	95% Confidence Interval Lower	Upper	df	t	p
(Intercept)	(Intercept)	0.48854	0.00950	0.46992	0.50716	232	51.435	< .001
Age	Age	-0.00289	5.29e-4	-0.00392	-0.00185	222	-5.456	< .001
Gender	Male - Female	-0.01114	0.01900	-0.04837	0.02609	232	-0.586	0.558
Age ✻ Gender	Age ✻ Male - Female	8.56e-4	0.00106	-0.00122	0.00293	222	0.809	0.419

atr_MD

Names	Effect	Estimate	SE	95% Confidence Interval Lower	Upper	df	t	p
(Intercept)	(Intercept)	6.72e-4	1.09e-5	6.51e-4	6.93e-4	243	61.666	< .001
Age	Age	3.57e-6	6.10e-7	2.37e-6	4.76e-6	238	5.850	< .001
Gender	Male - Female	8.45e-6	2.18e-5	-3.43e−5	5.12e-5	243	0.388	0.699
Age ✻ Gender	Age ✻ Male - Female	-4.29e−7	1.22e-6	-2.82e−6	1.96e-6	238	-0.352	0.725

atr_AD

Names	Effect	Estimate	SE	95% Confidence Interval Lower	Upper	df	t	p
(Intercept)	(Intercept)	0.00105	1.16e-5	0.00103	0.00108	232	90.724	< .001
Age	Age	3.30e-6	6.46e-7	2.03e-6	4.56e-6	222	5.101	< .001
Gender	Male - Female	-7.42e−6	2.32e-5	-5.29e−5	3.81e-5	232	-0.320	0.750
Age ✻ Gender	Age ✻ Male - Female	7.97e-7	1.29e-6	-1.74e−6	3.33e-6	222	0.617	0.538

Supplementary Table2. interaction between age and gender on the developmental trajectories of WM tracts

atr_RD

Names	Effect	Estimate	SE	95% Confidence Interval Lower	Upper	df	t	p
(Intercept)	(Intercept)	4.78e-4	1.22e-5	4.54e-4	5.02e-4	240	39.135	< .001
Age	Age	3.91e-6	6.82e-7	2.57e-6	5.25e-6	233	5.730	< .001
Gender	Male - Female	1.36e-5	2.44e-5	-3.42e−5	6.15e-5	240	0.558	0.577
Age ✻ Gender	Age ✻ Male - Female	-8.82e−7	1.36e-6	-3.56e−6	1.79e-6	233	-0.647	0.519

4. Age-related changes in schizotypy

Supplementary Table 3. interaction between age and gender on the developmental trajectories of schizotypy

Positive schizotypy

Names	Effect	Estimate	SE	95% Confidence Interval Lower	Upper	df	t	p
(Intercept)	(Intercept)	19.594	2.297	15.0923	24.0966	228	8.53	< .001
Age	Age	-0.710	0.128	-0.9610	-0.4595	216	-5.55	< .001
Gender	Male - Female	-9.947	4.589	-18.9411	-0.9528	227	-2.17	0.031
Avg(VocBlockDE)	Avg(VocBlockDE)	-0.379	0.160	-0.6921	-0.0663	242	-2.38	0.018
Age ✻ Gender	Age ✻ Male - Female	0.439	0.256	-0.0636	0.9416	215	1.71	0.088

Negative schizotypy

Names	Effect	Estimate	SE	95% Confidence Interval Lower	Upper	df	t	p
(Intercept)	(Intercept)	7.86765	1.6364	4.660	11.0749	228	4.8079	< .001
Age	Age	-0.11511	0.0912	-0.294	0.0636	217	-1.2628	0.208

Negative schizotypy

Names	Effect	Estimate	SE	95% Confidence Interval Lower	Upper	df	t	p
Gender	Male - Female	0.60385	3.2692	-5.804	7.0113	228	0.1847	0.854
Avg(VocBlockDE)	Avg(VocBlockDE)	-0.00496	0.1136	-0.228	0.2176	241	-0.0437	0.965
Age ✻ Gender	Age ✻ Male - Female	-0.06658	0.1827	-0.425	0.2916	215	-0.3644	0.716

Disorganized schizotypy

Names	Effect	Estimate	SE	95% Confidence Interval Lower	Upper	df	t	p
(Intercept)	(Intercept)	8.2205	1.4488	5.3810	11.06005	232	5.674	< .001
Age	Age	-0.1641	0.0809	-0.3226	-0.00566	222	-2.030	0.044
Gender	Male - Female	-6.9199	2.8948	-12.5935	-1.24623	231	-2.390	0.018
Avg(VocBlockDE)	Avg(VocBlockDE)	0.0277	0.0991	-0.1665	0.22197	234	0.280	0.780
Age ✻ Gender	Age ✻ Male - Female	0.3331	0.1621	0.0154	0.65084	220	2.055	0.041

Supplementary Figure 2. Age-related changes in schizotypy

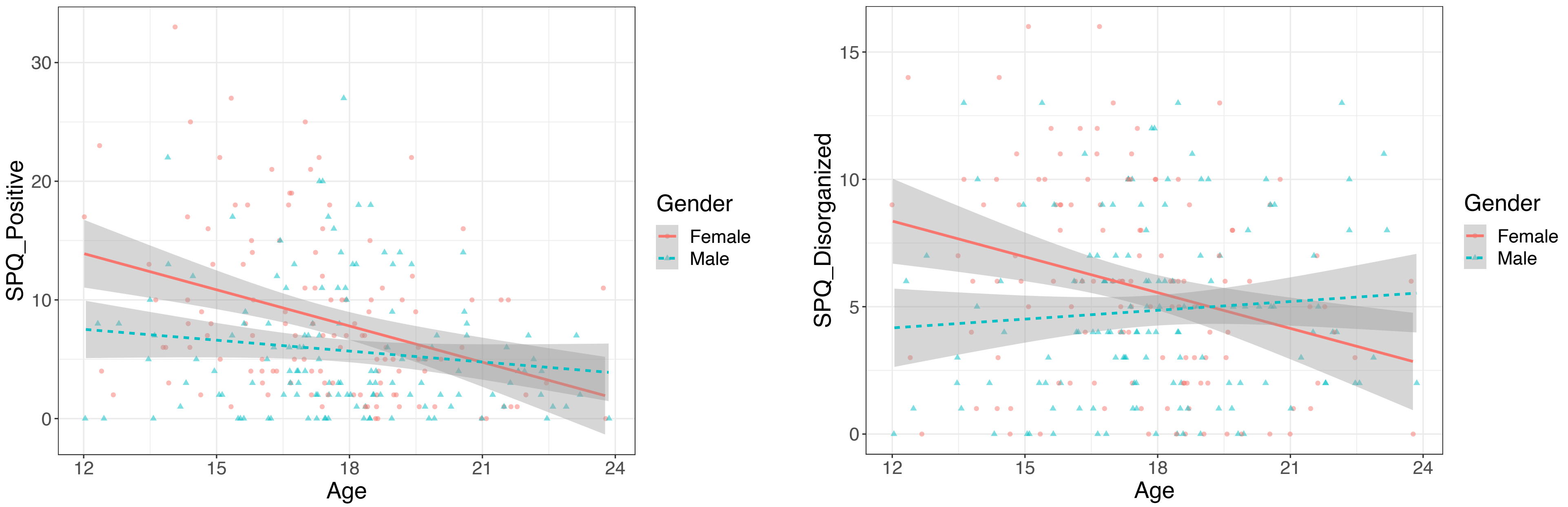

4. Interaction between age and diffusion parameters on the developmental trajectory of negative schizotypy

Supplementary Table 4. Interaction between age and diffusion parameters on the developmental trajectory of Negative schizotypy

Fixed Effects Parameter Estimates

				95% Confidence Interval				
**Names**	**Effect**	**Estimate**	**SE**	**Lower**	**Upper**	**df**	**t**	**p**
(Intercept)	(Intercept)	8.1153	1.856	4.478	11.7525	226	4.373	< .001
Age	Age	-0.1344	0.104	-0.338	0.0694	204	-1.292	0.198
fmajorFA_Avg	fmajorFA_Avg	-127.9893	68.327	-261.907	5.9287	183	-1.873	0.063
fminorFA_Avg	fminorFA_Avg	41.2543	73.278	-102.368	184.8763	183	0.563	0.574
atr_FA	atr_FA	149.0435	105.560	-57.851	355.9377	202	1.412	0.160
cab_FA	cab_FA	93.4434	58.089	-20.409	207.2954	170	1.609	0.110
ccg_FA	ccg_FA	-20.9068	68.790	-155.732	113.9186	194	-0.304	0.762
cst_FA	cst_FA	-143.7374	89.377	-318.913	31.4378	188	-1.608	0.109
ilf_FA	ilf_FA	-51.0226	90.163	-227.739	125.6942	184	-0.566	0.572
slfp_FA	slfp_FA	-103.2654	138.428	-374.580	168.0494	196	-0.746	0.457
slft_FA	slft_FA	226.0697	169.108	-105.377	557.5160	185	1.337	0.183
unc_FA	unc_FA	-137.8427	92.476	-319.091	43.4060	188	-1.491	0.138
Avg(VocBlockDE)	Avg(VocBlockDE)	0.0135	0.118	-0.217	0.2441	228	0.115	0.909
Gender1	Male - Female	-0.2163	0.813	-1.810	1.3773	110	-0.266	0.791
Internalizing		0.2263	0.0254	0.1766	0.2760	227.2	8.9167	< .001
Externalizing		0.0184	0.0284	-0.0372	0.0741	218.8	0.6492	0.517
Age ✻ fmajorFA_Avg	Age ✻ fmajorFA_Avg	7.5298	3.754	0.173	14.8871	176	2.006	0.046
Age ✻ fminorFA_Avg	Age ✻ fminorFA_Avg	-3.2667	4.180	-11.460	4.9267	180	-0.781	0.436
Age ✻ atr_FA	Age ✻ atr_FA	-7.5799	5.977	-19.295	4.1356	196	-1.268	0.206
Age ✻ cab_FA	Age ✻ cab_FA	-5.1280	3.223	-11.444	1.1881	160	-1.591	0.114
Age ✻ ccg_FA	Age ✻ ccg_FA	0.4423	3.746	-6.900	7.7843	178	0.118	0.906
Age ✻ cst_FA	Age ✻ cst_FA	7.3565	5.053	-2.546	17.2595	177	1.456	0.147

Fixed Effects Parameter Estimates

Names	Effect	Estimate	SE	95% Confidence Interval Lower	95% Confidence Interval Upper	df	t	p
Age ✻ ilf_FA	Age ✻ ilf_FA	3.5057	5.104	-6.498	13.5093	172	0.687	0.493
Age ✻ slfp_FA	Age ✻ slfp_FA	5.2516	7.762	-9.962	20.4654	189	0.677	0.500
Age ✻ slft_FA	Age ✻ slft_FA	-12.1517	9.616	-30.998	6.6945	166	-1.264	0.208
Age ✻ unc_FA	Age ✻ unc_FA	7.5383	5.184	-2.623	17.6997	175	1.454	0.148

note. fmaj represents the corpus callosum forceps major, fmin, the forceps minor, cst the corticospinal tract, slfp the superior longitudinal fasciculus parietal, slft the temporal, ilf the inferior longitudinal fasciculus, ccg the cingulum-cingulate gyrus bundle, cab the cingulum angular bundle, unc the uncinate fasciculus. SE stands for standard error.

4.b. Inclusion of covariates

In each analysis, sex was added as a covariate of no interest. In follow up analyses to significant results, we examined whether results would hold when controlling for current internalizing and externalizing scores as well as cognitive functioning (averaged Block Design and Vocabulary standardized scores). All covariates were entered in the model as standardized variables (mean-centred and scaled across sample).

All above-described results on the association between schizotypy and white matter tracts remained significant when the models were controlled for the additional covariates of internalizing and externalizing. While the significance of the model was slightly decreased, results remained significant in the same tracts. Follow up analysis was conducted with cognitive functioning as covariate to test whether it had an impact on trajectories. When the average between block design and vocabulary was additionally included in the model, all interaction and simple effects showed earlier remained significant.

5.a Supplementary analysis - Attrition differences

Out of the 102 that were included at T0 or T1, 25 dropped out of the longitudinal study (meaning they never came back at any other time point), while 77 of them came back for at least 1 of the other longitudinal timepoints. We compared the 2 groups (Group1, n=77: participants with return visits VS Group2, n=25: dropouts) on schizotypy dimensions, demographics, and mean FA measures within tracts. The two attrition groups did not differ significantly on any of the variables of interest.

Descriptives

	Attrition_Group	SPQ_Positive	SPQ_Negative	SPQ_Disorganized	Age	Avg(VocBlockDE)	YSR_Internalizing_TScore	YSR_Externalizing_TScore
N	Came back	77	77	77	77	77	77	77
	Drop Out	25	25	25	25	25	25	25
Mean	Came back	8.84	6.21	5.78	15.9	11.2	52.9	56.1
	Drop Out	8.56	6.32	5.72	16.8	10.7	53.8	54.9
SD	Came back	7.10	4.40	3.60	1.79	2.69	11.0	9.12
	Drop Out	6.84	4.79	4.19	1.82	1.81	9.17	10.3

Kruskal-Wallis

	χ^2	df	p	ε^2
SPQ_Positive	0.10950	1	0.741	0.00107
SPQ_Negative	0.07511	1	0.784	7.36e-4
SPQ_Disorganized	0.03493	1	0.852	3.42e-4
Age	4.45429	1	0.055	0.04367
fmajorFA_Avg	1.05039	1	0.305	0.01030
fminorFA_Avg	0.22155	1	0.638	0.00217
atr_FA	1.47545	1	0.224	0.01447
cab_FA	0.76222	1	0.383	0.00747
ccg_FA	0.47727	1	0.490	0.00468
cst_FA	0.00467	1	0.946	4.58e-5
ilf_FA	1.47545	1	0.224	0.01447
slfp_FA	0.22875	1	0.632	0.00224
slft_FA	0.09688	1	0.756	9.50e-4
unc_FA	0.94429	1	0.331	0.00926
Avg(VocBlockDE)	1.83934	1	0.175	0.01803
YSR_Internalizing_TScore	0.09475	1	0.758	9.29e-4
YSR_Externalizing_TScore	0.63062	1	0.427	0.00618

5.b. Supplementary analysis: Changes pf schizotypal scores along time interval of the study

Linear (t(170)=-5.686, p<0.001) and quadratic (t(168)=2.957, p=0.004) modelling of time had a significant effect on positive schizotypy (see below the quadratic representation). Linear (t(174)=-2.14, p=0.034) and quadratic (t(171)=2.15, p=0.033) modelling of time also had a significant effect on disorganized schizotypy (see below the quadratic representation). However, there was no significant effect of time on negative schizotypy.

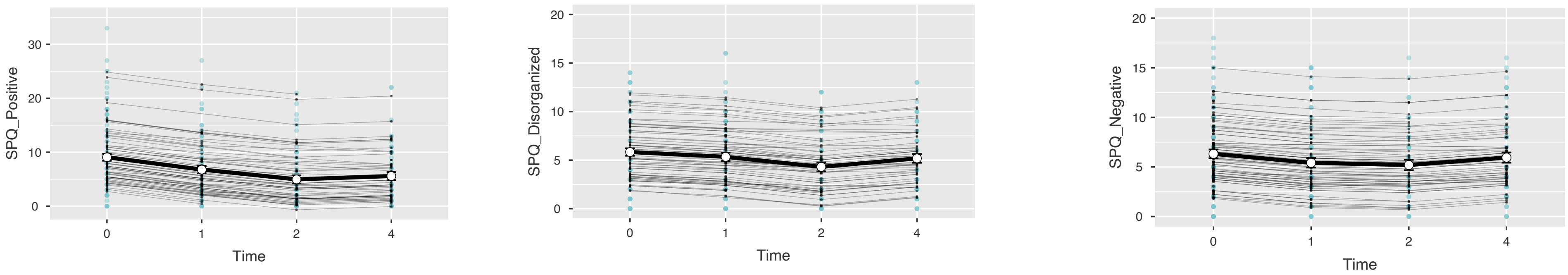

6.a. Age-related changes in schizotypy

We reported that both positive and disorganized schizotypal dimensions exhibited significant linear decreasing trajectories with age (between 12 and 24 y.o). Furthermore, the interaction between gender and age was significant only for the disorganized schizotypy trajectory; females showed a relatively steep linear decreasing trajectory, while males followed a steadier linear increasing trajectory. These results confirm previous findings suggesting that psychotic symptom-like features and disorganization decrease from late adolescence to older ages in both sexes [6,7] and add that the gradual decrease is already observable in late childhood/early adolescence. Although this reduction in schizotypal features was gradual in most cases, there was a different pattern for disorganization for male subjects who showed an increasing trajectory. The latter is against results from Bora and Baysan who found an increase in females' disorganized features at the end of adolescence, while our sample showed that females expressed higher disorganized features than males overall. It must be noted however that their study was not longitudinal and only compared age-group effects on schizotypy. In our case, we could expect males' disorganization to increase slightly during adolescence and decrease at older ages following a non-linear trajectory. However, our oldest participants were late adolescents, thus we would need longitudinal data on older participants to confirm this claim.

6.b Age-related changes in diffusion parameters

We observed in our sample of adolescents an association between the anterior thalamic radiation and age. The decrease in FA was associated with a significant increase in all other WM parameters (MD, AD, RD). The input from the thalamus during adolescence is known to be important for PFC circuit maturation in mice, and altering the transmission of this input leads to long-lasting impairments in the functioning of the PFC [8]. Thus, it seems that in our sample of adolescents, the normative maturation of the ATR is described

by an overall decrease in FA. Human studies have shown that the anterior thalamic radiation develops later in adolescence and adulthood with FA[9], which could explain why we do not observe the typical increase in FA in the ATR. Besides, the same authors showed that aging does not affect major white matter tract indices uniformly, with the part closer to the thalamus maturing earlier than the frontal part, thus we might only capture the overall development of the tract in our sample.

6.c Absence of results for negative schizotypy

It is not surprising that we could not observe any association between the negative dimension of schizotypy and neither DTI parameters, not DTI parameters * Age, as previous studies on schizophrenia patients did not find any correlations between negative symptoms and FA[10]. Importantly, in their study Lener et al [10] included schizophrenia patients who were unmedicated and not chronic, whereas other studies on chronic and medicated schizophrenia population have shown association between negative symptoms severity and FA [11,12]. Thus, we could hypothesize that negative features might only influence white matter integrity in the most clinical and chronic stages of the schizophrenia spectrum.

References

1. Cautionary Statement for Forensic Use of DSM-5. In: *Diagnostic and Statistical Manual of Mental Disorders, 5th Edition*. American Psychiatric Publishing, Inc; 2013. doi:10.1176/appi.books.9780890425596.744053

2. Isabel A Bordin, Marina M Rocha, Bahia L. Child Behavior Checklist (CBCL),Youth Self-Report (YSR) and Teacher's Report Form(TRF): an overview of the development of the original and Brazilian versions. *Cad Saúde Pública*. 2013;29(5):851-866. doi:10.1590/S0102-311X2013000500004

3. Mahr S, Petot JM, Camart N, Zebdi R. Structure factorielle et qualités psychométriques de la version française du questionnaire d'auto-description pour adultes (adult self-report) d'Achenbach et Rescorla. *Psychologie Française*. 2018;63(1):23-36. doi:10.1016/j.psfr.2016.12.001

4. Dale AM, Fischl B, Sereno MI. Cortical Surface-Based Analysis. :16.

5. Reuter M, Fischl B. Avoiding asymmetry-induced bias in longitudinal image processing. *NeuroImage*. 2011;57(1):19-21. doi:10.1016/j.neuroimage.2011.02.076

6. Bora E, Arabaci LB. Effect of age and gender on schizotypal personality traits in the normal population. *Psychiatry and Clinical Neurosciences*. 2018;63(5):663-669. doi:10.1111/j.1440-1819.2009.02011.x

7. Karamaouna P, Zouraraki C, Giakoumaki SG. Cognitive Functioning and Schizotypy: A Four-Years Study. *Front Psychiatry*. 2021;11:613015. doi:10.3389/fpsyt.2020.613015

8. Benoit LJ, Holt ES, Posani L, et al. Adolescent thalamic inhibition leads to long-lasting impairments in prefrontal cortex function. *Nat Neurosci*. 2022;25(6):714-725. doi:10.1038/s41593-022-01072-y

9. Lynch KM, Cabeen RP, Toga AW, Clark KA. Magnitude and timing of major white matter tract maturation from infancy through adolescence with NODDI. *NeuroImage*. 2020;212:116672. doi:10.1016/j.neuroimage.2020.116672

10. Lener MS, Wong E, Tang CY, et al. White Matter Abnormalities in Schizophrenia and Schizotypal Personality Disorder. *Schizophrenia Bulletin*. 2015;41(1):300-310. doi:10.1093/schbul/sbu093

11. Nakamura K, Kawasaki Y, Takahashi T, et al. Reduced white matter fractional anisotropy and clinical symptoms in schizophrenia: A voxel-based diffusion tensor imaging study. *Psychiatry Research: Neuroimaging*. 2012;202(3):233-238. doi:10.1016/j.pscychresns.2011.09.006

12. Wolkin A, Choi SJ, Szilagyi S, Sanfilipo M, Rotrosen JP, Lim KO. Inferior Frontal White Matter Anisotropy and Negative Symptoms of Schizophrenia: A Diffusion Tensor Imaging Study. *AJP*. 2003;160(3):572-574. doi:10.1176/appi.ajp.160.3.572
